# Incremental diagnostic role of cardiac MRI in young-middle aged patients with high-grade atrio-ventricular block

**DOI:** 10.1186/1532-429X-18-S1-O127

**Published:** 2016-01-27

**Authors:** Anna Baritussio, Amardeep Ghosh Dastidar, Nauman Ahmed, Jonathan Rodrigues, Antonio Frontera, Chris B Lawton, Daniel Augustine, Elisa McAlindon, Chiara Bucciarelli-Ducci

**Affiliations:** grid.410421.20000000403807336Bristol NIHR Cardiovascular Biomedical Research Unit, Bristol Heart Institute, Bristol, UK

## Background

Atrio-ventricular (AV) block is a common brady-arrhythmia in the elderly, but is a rare event in young or middle-aged adults, often leading to pacemaker implantation without further investigation, though underlying aetiology influences both treatment strategies and prognosis. Cardiovascular magnetic resonance (CMR) has the potential to identify an underlying aetiology for AV block, over and above transthoracic echocardiogram (TTE), which is offered as the first imaging technique. We sought to assess the diagnostic additive role of CMR in young and middle aged adults (18-60 years) with high-grade AV block and to determine which findings on CMR best predict clinical impact.

## Methods

We retrospectively analysed the CMR registry from a tertiary centre in the South-West of England to collect data on consecutive high-grade AV block patients (18-60 yrs) referred for CMR between September 2012 to July 2015. High-grade AVB was defined as the evidence of Mobitz II 2^nd^ degree or complete AVB on resting electrocardiogram (ECG). Patients underwent TTE and a comprehensive CMR protocol (including long and short axis cines, and late gadolinium enhancement, LGE, imaging). A change in diagnosis was defined as CMR findings leading to a new diagnosis compared to a multi-parametric pre-CMR diagnosis (clinical data, ECG and TTE).

## Results

We identified 31 patients with AV block (17 male, 55%) with a mean age of 43 ± 11 years (IQR 33-53 years). CMR was diagnostic in all but 2 patients (7%): an ischemic heart disease was found in 3 (10%) patients, non-ischemic heart disease in 11 (35%), of which 3 had infiltrative cardiomyopathy, and a structurally normal heart in 15 (48%). As compared to pre-CMR diagnosis, CMR findings led to a change in diagnosis in 45% of patients. In a multivariate model adjusting for demographic and CMR characteristics, only LGE was a significant independent predictor of the underlying diagnosis (p 0.4, 95% CI 1.07-62.1) (Table 1).

**Table 1:** Predictors of clinical impact

	Sig.	Exp (B)	95% C.I. for EXP (B)	
			Lower	Upper
Gender	0.184	4.226	0.505	35.383
Age	0.592	1.023	0.941	1.112
LVEF	0.081	1.230	0.975	1.552
LViEDV	0.253	1.019	0.986	1.053
LViSV	0.814	1.010	0.932	1.094
RVEF	0.209	0.847	0.654	1.098
LGE	0.042	8.174	1.075	62.139

Multivariable associations of demographic and CMR characteristics with subsequent clinical impact. LVEF, left ventricular ejection fraction; LViEDV, left ventricular indexed end-diastolic volume; LViSV, left ventricular indexed stroke volume; RVEF, right ventricular ejection fraction; LGE, late gadolinium enhancement.

## Conclusions

Management of AVB in young is challenging. Our study highlights that CMR can lead to a new diagnosis in almost half of patients. LGE was the only significant independent predictor of a new diagnosis compared to other imaging characteristics like biventricular ejection fraction and volumes. CMR should be included in the diagnostic work up of young patients with high grade AVB.

